# Analysis of Adhesion Molecules and Basement Membrane Contributions to Synaptic Adhesion at the *Drosophila* Embryonic NMJ

**DOI:** 10.1371/journal.pone.0036339

**Published:** 2012-04-30

**Authors:** Andre Koper, Annette Schenck, Andreas Prokop

**Affiliations:** 1 Faculty of Life Sciences, Wellcome Trust Centre for Cell-Matrix Research, Manchester, United Kingdom; 2 Department of Human Genetics, Nijmegen Centre for Molecular Life Sciences, Donders Institute for Brain, Cognition and Behaviour, Radboud University Nijmegen Medical Centre, Nijmegen, The Netherlands; VIB and KU Leuven, Belgium

## Abstract

Synapse formation and maintenance crucially underlie brain function in health and disease. Both processes are believed to depend on cell adhesion molecules (CAMs). Many different classes of CAMs localise to synapses, including cadherins, protocadherins, neuroligins, neurexins, integrins, and immunoglobulin adhesion proteins, and further contributions come from the extracellular matrix and its receptors. Most of these factors have been scrutinised by loss-of-function analyses in animal models. However, which adhesion factors establish the essential physical links across synaptic clefts and allow the assembly of synaptic machineries at the contact site *in vivo* is still unclear. To investigate these key questions, we have used the neuromuscular junction (NMJ) of *Drosophila* embryos as a genetically amenable model synapse. Our ultrastructural analyses of NMJs lacking different classes of CAMs revealed that loss of all neurexins, all classical cadherins or all glutamate receptors, as well as combinations between these or with a Laminin deficiency, failed to reveal structural phenotypes. These results are compatible with a view that these CAMs might have no structural role at this model synapse. However, we consider it far more likely that they operate in a redundant or well buffered context. We propose a model based on a multi-adaptor principle to explain this phenomenon. Furthermore, we report a new CAM-independent adhesion mechanism that involves the basement membranes (BM) covering neuromuscular terminals. Thus, motorneuronal terminals show strong partial detachment of the junction when BM-to-cell surface attachment is impaired by removing Laminin A, or when BMs lose their structural integrity upon loss of type IV collagens. We conclude that BMs are essential to tie embryonic motorneuronal terminals to the muscle surface, lending CAM-independent structural support to their adhesion. Therefore, future developmental studies of these synaptic junctions in *Drosophila* need to consider the important contribution made by BM-dependent mechanisms, in addition to CAM-dependent adhesion.

## Introduction

Neuronal synapses are fascinating and complex asymmetric cell junctions, performing and regulating information transfer as a key feature of nervous system function. During synapse formation, two cells of distinct nature have to cooperate and form a very close connection at which complex machineries of transmitter release and reception assemble in precise alignment across the synaptic cleft. Cell adhesion molecules (CAMs) are believed to play central roles in this process [1]–[7]. They are essential during the process of axon guidance and target recognition, and they can establish and maintain synaptic cell junctions in cell culture [8], [9]. Furthermore, through signalling processes or physical recruitment of protein scaffolds at their cytoplasmic domains, CAMs are believed to direct the assembly of pre- and postsynaptic machineries, which can be linked together across the synaptic cleft through the extracellular domains of these CAMs [7], [10]. Therefore, understanding the precise role of CAMs lies at the heart of our quest for understanding the process of synapse formation and maintenance with implications for a wide range of neurodevelopmental, psychiatric and neurodegenerative disorders [11], [12].

Different classes of CAMs localise to synapses, such as cadherins, protocadherins, neuroligins, neurexins, integrins, and immunoglobulin adhesion proteins. Many of these have been scrutinised by loss-of-function analyses in animal models. However, we still lack clear examples of CAMs which are required *in vivo* to mediate the essential physical links across synaptic clefts or induce the intracellular assembly processes of the synaptic machinery [2], [3], [5]. Analyses are hampered by the fact that CAMs play numerous roles during neuronal circuit and synapse formation and maintenance. Thus, synapse loss can be caused by aberrant patterns of neurogenesis or apoptosis, defects in neural migration or axonal guidance, or the regulated retraction of synaptic terminals during plastic circuit remodelling [13]–[15]. As a further complication, many CAMs perform signalling functions which might regulate other structural or adhesion proteins [16], [17]. However, the main obstacle to experimental analyses is the lack of prominent synaptic phenotypes upon loss of CAM functions [2], [3], [5]. They usually become more apparent only when taking out a number of genes in parallel (e.g. neuroligin triple knockout mice, or *en bloc* deletion of 22 of the ca. 60 protocadherin genes) [13], [18]. Therefore, synaptic CAMs are believed to form a redundant adhesion code at synapses, the decryption of which remains a major challenge.

Loss of function phenotypes where pre- and postsynaptic terminals are in place but display partial or complete junctional detachment or changes of synaptic cleft widths have rarely been reported [2], [3], [5]. Reported phenotypes of this kind were primarily identified at the vertebrate neuromuscular junction (NMJ) and relate primarily to mechanisms involving the extracellular matrix. Vertebrate NMJs are cholinergic synaptic terminals with an unusually wide cleft of 60–70 nm containing a basal lamina. Accordingly, their differentiation is largely dependent on extracellular matrix and matrix receptors [19]. For example, type XIII collagen-deficient mice display a partial detachment of presynaptic motoraxon terminals from postsynaptic sites on muscles [20]. NMJs deficient for junctional laminins lack presynaptic active zones, and the area of contacts between motoraxon terminals and muscles is severely reduced and substituted by invading processes of Schwann cells [21]. This phenotype is likely due to adhesive functions of these laminins, which physically link to transmembrane receptors but also repel glia cells [19]. Also hippocampal synapses in these laminin-deficient mice show irregular spacing of their synaptic clefts [22].

Like vertebrate NMJs, *Drosophila* NMJs are easy to identify and visualise due to their characteristic location on peripheral muscles and represent a promising paradigm for the investigation of synaptic adhesion [23], [24]. In contrast to vertebrates, *Drosophila* NMJs share a number of features with excitatory central synapses of vertebrates [23], [24]. They have a narrow synaptic cleft of 15–20 nm, they are glutamatergic and display conserved molecular components in their pre- and postsynaptic machineries [25], [26]. Accordingly, *Drosophila* NMJs display a range of evolutionarily conserved synaptic CAMs, such as cadherins, neurexins, integrins, syndecan and dystroglycan [6], [27]. Notably, loss-of-function analyses of these factors are enormously facilitated in the fly by the far lower number of genes encoding adhesion proteins and by the large pool of readily available mutations and genetic tools (www.flybase.org). Furthermore, loss of Laminin A in *Drosophila* embryos causes significant reduction in NMJ adhesion, providing an example of a factor that might directly contribute to the establishment of adhesion at the *Drosophila* NMJ [28].

Here we used the embryonic *Drosophila* NMJs as a model to analyse the contributions of CAMs to the structural differentiation of synapses. Using primarily electron microscopy (EM), we analysed potential structural defects at NMJs with combined loss of different sets of synaptic CAMs. We found that loss of all neurexins, all classical cadherins or all glutamate receptors at NMJ, as well as several combinations between these or with Laminin deficiency, fail to reveal structural phenotypes. This suggests that these CAMs are either not important for the structural development of this model synapse or operate as a highly redundant genetic network. We report that partial detachment of NMJs upon loss of Laminin A is caused by mechanisms independent of CAMs and involves mechanical support provided by basement membranes (BM) that cover neuromuscular terminals. Therefore, we propose impairing BM adhesion as a valuable strategy for future studies that address CAM functions at embryonic *Drosophila* NMJs.

## Results

### A strategy for the functional analysis of synaptic adhesion molecules

**Figure 1:**
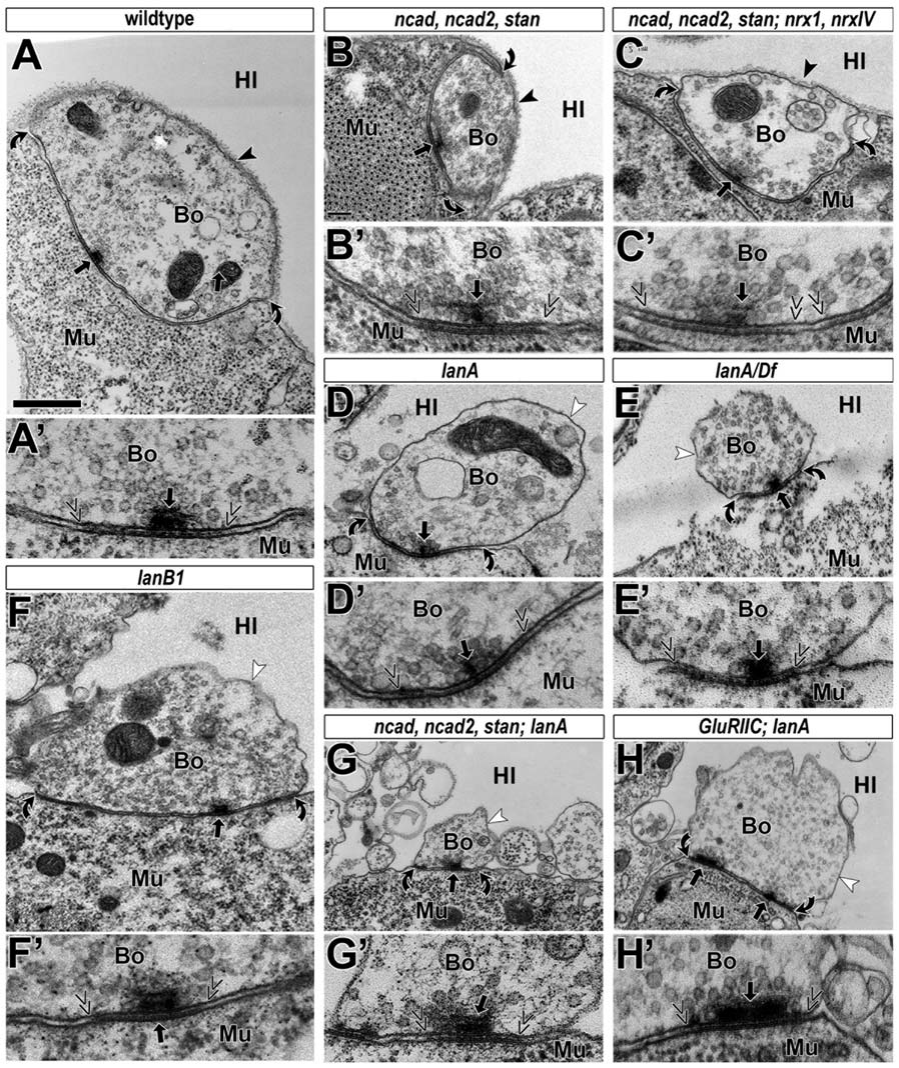
Examples of ultrastructural phenotypes of doubly or multiply mutant embryos. Images of neuromuscular bouton profiles (A–H) and close-ups of their respective synapses (A′–H′) in late stage 17 embryos of wildtype (**A**) or animals carrying the following mutant allele combinations: *CadN-CadN2(ΔN14)*, *stan^192^* in homozygosis (**B**), *CadN-CadN2(Δ14)*, *stan^192^*; *Nrx-1^Δ83^*, *Nrx-IV^4304^* in homozygosis (**C**), *lanA^9.32^*/*lanA^9.32^* (**D**), *lanA^9.32^*/*Df(3L)Excel8101* (**E**), *lanB1^DEF^*/*lanB1^DEF^* (**F**), *CadN-CadN2(ΔN14)*, *stan^192^; lanA^9.32^* in homozygosis (**G**), *GluRIIC^1^; lanA^9.32^* in homozygosis (**H**; see further info on GluRIIC in the legend of Fig. 2). Symbols and abbreviations are consistently used for all micrographs throughout this manuscript: Bo, presynaptic bouton; Mu, postsynaptic muscle; Hl, haemolymph; black arrows, active zones; arrow heads, BMs; curved arrows, demarcate neuromuscular contacts; double chevrons, demarcate synapses; white arrow heads, cell surfaces lacking BMs. No changes in adhesion index or synaptic structure were detected in A–C, whereas the adhesion index in D–H was changed from ∼50% to ∼25% in the absence of any further structural changes (quantified in Fig. 2A–D). Scale bar in A represents 500 nm in A–H and 200 nm in A′–H′.

In order to assess the relevance of CAMs for adhesion and the structural differentiation of *Drosophila* NMJs, we analysed the effects of loss of function mutations functionally removing candidate CAMs. Since many of these mutations are embryonic lethal, we restricted our analyses to late stage 17 embryos which display fully differentiated and functional neuromuscular terminals. In contrast to larval NMJs, which are submerged beneath the muscle surface and surrounded by a reticular network of muscle membrane infoldings, synaptic adhesion at embryonic NMJs is far easier to interpret in that boutons are located on the surfaces of muscles and do not yet display infoldings of postsynaptic muscle membranes (Fig. 1A) [23], [24].

To have the appropriate resolution for our studies, we carried out EM analyses focussing on several prominent structural features of NMJs: the adhesion index (the percentage of the circumference of active zone-bearing boutons that is in contact with muscle membrane; between curved arrows in Fig. 1) [28], the integrity of active zones including docked and surrounding synaptic vesicles (black arrows), the width of the synaptic cleft and presence of electron-dense material within it (between double-chevrons), and the integrity and adhesion of BMs (back arrow heads). We decided to focus these analyses primarily on neurexins, classical cadherins and seven-transmembrane cadherins, since they represent classical synaptic adhesion protein classes, for which we saw a realistic chance to genetically remove all family members simultaneously in the same animals. We also included removal of ionotropic glutamate receptors (GluRs) in our analyses, based on the rationale that they might act as unconventional adhesion receptors or play key roles in synaptic assembly processes. In contrast, the immunoglobulin superfamily was excluded, since it is enormously complex. Many members are expressed in the CNS [29] (www.flybase.com), which makes the goal of ablating this CAM class difficult to achieve.

### Neurexins have no obvious structural requirements at embryonic *Drosophila* NMJs

**Figure 2:**
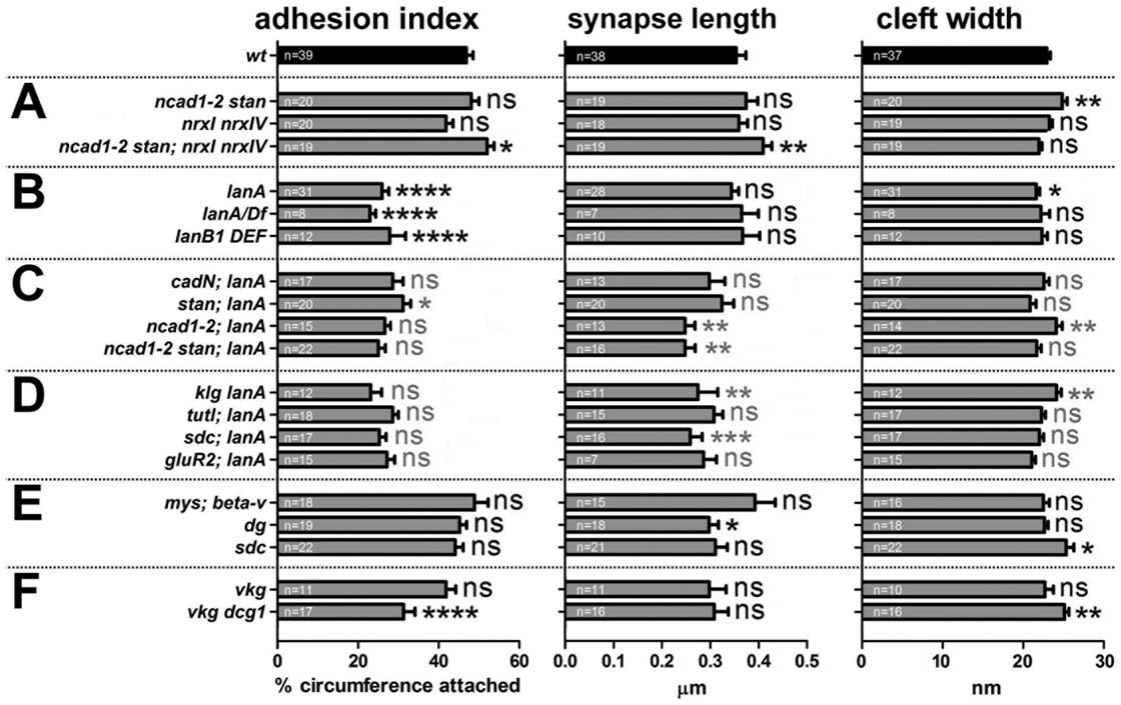
Quantifications of ultrastructural NMJ phenotypes. Embryonic NMJ boutons displaying active zones (arrows in Figs. 1, 4 and 5) were measured. Genotypes are grouped into combinations of cadherins and neurexins (**A**), Laminin-deficient conditions (**B**), combinations of *lanA^9.32^* with loss of cadherins (**C**), loss of other potential adhesion factors (as explained below) in combination with *lanA^9.32^* (**D**), loss of classical laminin receptors (**E**), and collagen type IV-deficient conditions (**F**). The following parameters were analysed: *“adhesion index”*, the percentage of the circumference of active zone-bearing boutons that is in contact with muscle membrane (between curved arrows in Figs. 1, 4 and 5); “*synapse length*”, mean length of electron dense cleft material known to indicate synapse diameter (between double chevrons in Figs. 1, 4 and 5); “*cleft width*”, mean distance between pre- and postsynaptic membranes at synapses. *Bars* represent mean ± standard error of the mean; *n*, number of assessed NMJ boutons sampled from at least 5 embryos, respectively; *asterisks* indicate statistical significances as compared to wt (black asterisks) or lanA (grey asterisks; *, P≤0.1; **, P≤0.01; ***, P≤0.001; ****, P≤0.0001 according to Mann Whitney tests). Additional information on included CAMs not explained in the main text: the immunoglobulin adhesion receptor Klingon is suggested to express potential synaptic functions [88], [89]; the immunoglobulin adhesion receptor Turtle acts as a homophilic adhesion factor in S2 cell assays which has demonstrated neuronal phenotypes *in vivo* [91], [92], [104], [105]; the transmembrane heparan sulfate proteoglycan Syndecan (Sdc) might act as a CAM by serving as a ligand for the motorneuronal receptor Lar (Leukocyte-antigen-related-like, a close homolog of avian protein tyrosin phosphatase ó) [106]–[108].

Neurexins are presynaptic transmembrane receptors rich in EGF-like and Laminin G-like domains which are sufficient to mediate synapse formation in cell culture through heterophilic interaction with postsynaptic neuroligins [9], [30]. In *Drosophila*, the neurexin superfamily is represented by two genes, Neurexin 1 (Nrx-1) and Neurexin IV (Nrx-IV), both of which were described to be localised and functionally required at NMJs [31], [32]. Previous ultrastructural studies revealed no obvious NMJ phenotypes in *Nrx-IV^4304^* mutant embryos [33] and a mostly normal appearance of NMJs in Nrx-1 mutant embryos [34], whereas Nrx-1 mutant larvae displayed reduced synapse numbers per area in the larval brain [35] and an increase in synapse numbers and sizes in neuromuscular boutons accompanied by focal invaginations of the presynaptic membrane [32]. To address the role of the Neurexin family in NMJ adhesion, we combined the two loss-of-function mutant alleles (*Nrx-1^Δ83^* snd *Nrx-IV^4304^*) onto one chromosome and carried out a qualitative and quantitative analysis. However, ultrastructural analyses of NMJs in *Nrx-1^Δ83^, Nrx-IV^4304^* double-mutant embryos did not reveal any changes in the adhesion index, the synaptic diameter and the synaptic cleft width (quantified in Fig. 2A), and active zones showed no obvious structural aberrations (not shown, but see Fig. 1C). We also did not find supporting evidence for the previously reported occasional misalignment of pre- and postsynaptic specialisations in the Nrx-1 single mutant [34]. Potential phenotypes are unlikely to be masked by maternally contributed protein, since previous studies of zygotic mutant embryos showed that both proteins are absent at mid-embryonic stages [32]–[34]. Therefore, combined lack of both *Drosophila* neurexins is insufficient to cause structural or loss of adhesion phenotypes at embryonic NMJs.

### Loss of two Cadherin classes has no obvious impact on NMJ structure

**Figure 3:**
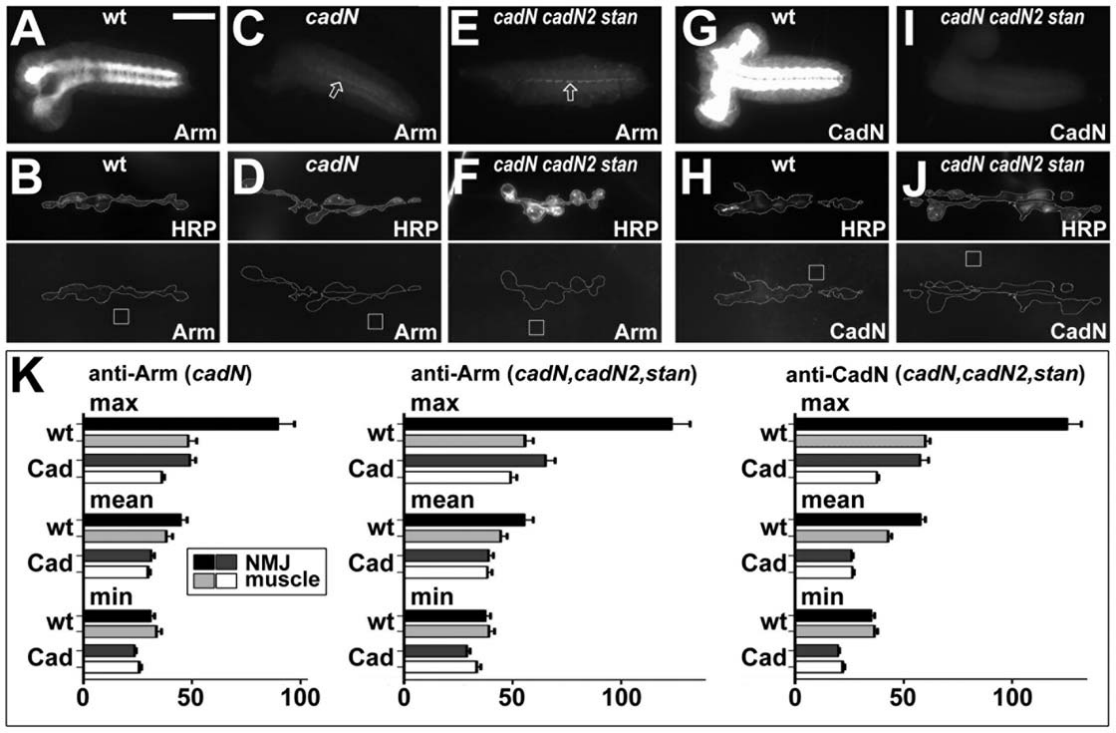
Immunohistochemical characterisation of synaptic cadherins. **A–J**) CNSs (top row) or NMJs (bottom rows) of wildtype embryos (wt), or of embryos homozygous for *cadN^M19^* (*cadN*) or *CadN-CadN2(Δ14)*, *stan^192^* (cadN cadN2 stan), stained either for Armadillo/ß-Catenin (A–F) or for CadN (G–J); HRP staining serves as a general indicator of neuronal membranes. **K**) Fluroresence measurements of anti-Armadillo/ß-Catenin and anti-CadN stainings at NMJs of the above embryos (as indicated on top of each graph) are represented as absolute values on the standard scale for pixel intensities (0 = no signal; 255 = full signal); black and dark grey bars represent Armadillo/ß-Catenin or CadN levels at the NMJ (demarcated by white line in above images), light grey and white bars represent background staining on neighbouring muscle membranes (demarcated by white boxes in above images); bars are grouped into maximal, mean and minimal values of the measurements. Scale bar in A represents 12 µm in top row and 900 nm in the second and third rows.

Classical cadherins (i.e. those associating with catenins through their cytoplasmic domains) and protocadherins are highly enriched at vertebrate synapses [36], [37]. Amongst the 17 reported cadherin genes in *Drosophila*, there are no protocadherins and only three classical cadherins: E-Cadherin (Shotgun, Shg), Cadherin-N (CadN) and Cadherin-N2 (CadN2/CG7527) [38]–[40]. Of these, Shg expression in the CNS seems to be restricted to midline glia cells in the embryonic CNS, extending to neural precursor cells only at larval stages [41]–[44]. In contrast, CadN has reported roles during pathfinding in the embryonic CNS, and both CadN and CadN2 contribute to synaptic targeting and morphology in the visual system of the fly [40], [45]–[47]. Loss of CadN staining in *CadN^M19^* null mutant embryos was demonstrated previously [45], and we confirmed that CadN was likewise abolished in the CNS and at NMJs of embryos carrying the *cadN1-2(Δ14)* deficiency which jointly removes CadN and CadN2 (Fig. 3I–K). Furthermore, ß-catenin (Armadillo, Arm; a reliable indicator for classical cadherin function) [43], [44], was suppressed below detectable levels in the neuropile (the CNS compartment containing the synapses) and at NMJs of embryos lacking CadN alone or in combination with CadN2 (Fig. 3C–F, K). Some Arm staining remained in midline glia cells, consistent with the expression of E-cadherin in these cells (open arrows in Fig. 3C, E) [42]. Although these findings suggest that CadN might be the only classical cadherin at synaptic contacts in *Drosophila* embryos, we nevertheless used *cadN1-2(Δ14)* double mutant embryos for our EM analyses. However, no ultrastructural phenotypes were discovered in these embryos at late stage 17 (quantified in Fig. 2A).

A further cadherin reported to mediate homophilic cell adhesion, to localise to NMJs and to regulate their morphology is Starry night/Flamingo (Stan) [48], [49]. Stan is the only seven-transmembrane protein with extracellular cadherin repeats in *Drosophila* and has three close mammalian homologues called CELSR1-3 [38], [50]. We used the *stan^192^* loss-of-function mutation and combined it with the *cadN1-2(Δ14)* deficiency deleting both CadNs. However, even these triple mutant embryos failed to reveal any obvious structural NMJ phenotypes when analysed by EM (Figs. 1B and 2A). To further challenge synaptic adhesion at NMJs, we generated mutant stocks in which loss of classical and seven-transmembrane cadherins was combined with loss of neurexins in the same animals. This fivefold homozygous mutant constellation [*cadN1-2(Δ14), stan^192^*; *nrx-I^Δ83^, nrx-IV^4304^*] was a unique constellation removing three classes of CAMs from *Drosophila* NMJs in parallel, yet synapses showed normal ultrastructure (Figs. 1C and 2A). Therefore, Cadherins and Neurexins are dispensable for adhesion and synaptic structure at embryonic NMJs.

### Laminin A is required for NMJ adhesion

An alternative explanation for the lack of adhesion phenotypes could be that CAM-independent forces contribute to NMJ adhesion. A potential variable could be BMs which cover synaptic terminals and closely adhere to non-attached surfaces of presynaptic terminals and muscles (black arrow heads in Fig. 1). We reasoned that these BMs might tie presynaptic terminals onto muscle surfaces, thus masking adhesive defects upon loss of CAMs in our analyses. In strong support of this hypothesis, loss of the BM constituent protein Laminin A was reported to cause severe BM and partial NMJ detachment [28]. We confirmed these results in embryos carrying the loss-of-function mutant allele *lanA^9.32^* either in homozygosis (*lanA^9.32^/lanA^9.32^*) or over a deficiency (*lanA^9.32^/Df(3L)Excel8101*). These mutant embryos showed severe absence of BMs from cellular surfaces, and a reduction in the adhesion index of neuromuscular boutons from ∼50% in wildtype to ∼25% in LanA-deficient embryos (Figs. 1D, E and 2B). To further validate Laminin as the cause for the observed phenotype, we analysed *lanB1^DEF^* mutant embryos which lack the laminin ß-chain LanB1. LanB1 is an essential constituent of the two existing Laminin A and W isoforms in *Drosophila* which only differ in their α-chains (encoded by the *lanA* and *wing blister* genes) [51]. Accordingly, our EM analyses of *lanB1^DEF^* mutant embryos revealed very similar phenotypes to those observed in *lanA^9.32^* (Figs. 1F and 2B), confirming the important role of Laminin A at NMJs and suggesting that Laminin W makes no obvious additional functional contributions to NMJ and BM adhesion. Notably, neither *lanA* nor *lanB1* mutant conditions affected other structural features of NMJs, such as the orderly appearance of active zones, synaptic vesicles or structured extracellular dense material in the synaptic cleft (quantified in Fig. 2B) [24].

We reasoned that these Laminin A-dependent adhesion mechanisms could be CAM-independent and might therefore mask potential phenotypes caused by loss of CAM functions. To test this hypothesis, we combined loss of CAM function with the *lanA^9.32^* mutant allele to create double, triple and quadruple homozygous mutant animals. Unfortunately, combinations of Nrx-1 and Nrx-IV with the *lanB1^DEF^* mutant allele severely affected early embryonic development, potentially indicating interesting functional links between Laminins and Neurexins. However, at the time of our studies, this genetic combination did not provide us with the biological material required for the intended NMJ analysis. In contrast, embryos with combined loss of Cadherins and Laminin A [i.e. *CadN^M19^; lanA^9.32^* or *cadN1-2(Δ14); lanA^9.32^* or *cadN1-2(Δ14)*, *stan^192^; lanA^9.32^*] developed to mature stages. However, their analysis revealed no enhancement over *lanA^9.32^* single mutant embryos in any of the assessed adhesive and structural features, although there is a slight tendency to have smaller synapse diameters (Figs. 1G and 2C). Finally, we tested GluRs as another class of molecules which can be entirely removed from *Drosophila* NMJs [52], based on the rationale that transmembrane channel proteins can have adhesive roles (e.g. calcium channels at the vertebrate NMJ) [53], [54]. Furthermore, *Drosophila* GluRs have been shown to contribute to molecular assembly processes at NMJs and their absence causes morphogenetic phenotypes at the light microscopic level [52], [55]. However, embryos carrying the *GluR2C^1^* mutation (removing all GluRs at the embryonic NMJ) failed to enhance the Laminin A-deficient phenotypes. Even dense material in the synaptic cleft was still visible (Figs. 1H and 2D), consistent with previous reports that pre- and postsynaptic markers still localise normally at NMJs of these mutant embryos [55].

Therefore, even when using Laminin deficiency as a sensitised background, loss of classical and seven-transmembrane cadherins or of GluRs at embryonic NMJs failed to reveal any obvious structural phenotypes, suggesting that they are either of low structural significance or display high functional redundancy in the context of NMJ structure (see Discussion). On the other hand, these experiments confirmed that Laminin A-deficient embryos display highly significant and reproducible reductions of adhesion indices at NMJs.

### Loss of classical Laminin receptors fails to reproduce the Laminin A-deficient adhesion phenotypes

We next aimed to understand the mechanism through which Laminin A contributes to NMJ adhesion. We reasoned that Laminin A could be required in the neuromuscular cleft, either by directly mediating adhesion, or through regulating the function of other CAMs. Alternatively, it could be required outside the neuromuscular cleft and mediate the cellular attachment of BMs, thus tying boutons onto muscle surfaces. To distinguish between these possibilities, we first aimed to identify the Laminin A receptors required in this context. Classical laminin receptors in vertebrates are integrins, dystroglycan and syndecans, and the same is true for their *Drosophila* homologues [51], [56]. The *Drosophila* genome encodes one Syndecan (Sdc), one Dystroglycan (Dg), two ß- (Myospheroid/Mys and ß^ν^ integrin/ßInt-ν) and five α-integrin subunits [51]. We tested whether loss of function of these receptors would resemble loss of Laminin A phenotypes.

**Figure 4:**
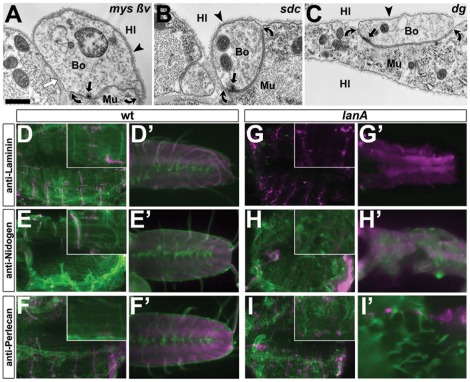
Exploring molecular mechanisms of Laminin A-dependent BM attachment. Images of neuromuscular bouton profiles (A–C) in late stage 17 embryos carrying the following mutant allele combinations: *mys^XG43^; ßInt-v^1^* in homozygosis (**A**), *Sdc^97^*/*Sdc^23^* (**B**), *Dg^043^*/*Dg^086^* (**C**); no changes in adhesion indices were detected (statistical validation in Fig. 2E); white arrows indicate pseudo-cell contacts separated by BMs, all other symbols as explained in Fig. 1. **D–E′**) Tissues of late stage 17 wildtype (left) or *lanA^9.32^* mutant embryos (right): D–I) show flat-dissected whole body preparations (insets show the ventro-longitudinal muscles VL1-4) [109]; D′–H′) show isolated CNSs; I′ shows a close up of a flat dissected embryo; preparations are immuno-stained against Laminin, Nidogen or Perlecan (in green; as indicated on the left) in combination with anti-HRP labelling neuronal tissues (magenta). Perlecan and Nidogen are still present within fragmented BMs of *lanA^9.32^*-mutant embryos. Scale bar in A represents 600 nm in A, 200 nm in B and C, 80 µm in D–I (insets 2.5 fold enhanced), and 30 µm D–I′.

When analysing *Dg^043^/Dg^086^* and *Sdc^97^/Sdc^23^* null mutant embryos we found no obvious defects of NMJ and BM adhesions, nor did we see any other obvious structural aberrations at synapses (Figs. 2E and 4B, C). Genetic interference with integrins containing the ß-subunit Myospheroid (Mys) has previously been shown to mediate Laminin A-independent BM attachment only at scattered focal contacts on muscle surfaces, but not to be required for general cell surface attachment of BMs [28]. Therefore, we included ßInt-ν into our studies which is known to execute functions redundant to Mys in other contexts [57], [58] and to be present at NMJs [59]. However, also in *mys^XG43^; ßInt-ν* double-null mutant embryos we found no changes of BM or NMJ attachments (Figs. 2E and 4A). Therefore, none of the classical laminin receptors classes alone mediates the laminin-dependent BM adhesion.

### Perlecan and Nidogen localise to BMs in the absence of Laminin A

**Figure 5:**
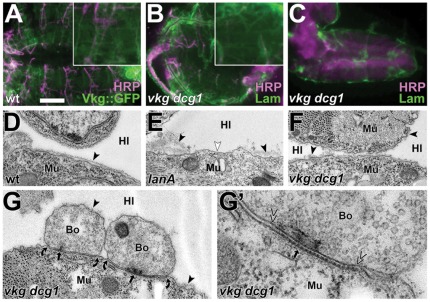
Loss of type IV collagens reproduces NMJ detachment phenotypes without affecting Laminin A localisation. **A–C**) Flat-dissected whole body preparations (A, B) and a CNS (C) of wildtype (wt) or *Df(2L)Exel7022* homozygous mutant embryo (*vkg dcg1*) at late stage 17, immuno-stained against GFP or Laminin and with the neuronal marker anti-HRP (as indicated bottom right); insets in A and B show the ventro-longitudinal muscles VL1-4; Vkg::GFP localises to BMs in wildtype embryos and Laminin is present in fragmented BMs of *Df(2L)Exel7022* embryos. **D–F**) Ultrastructural images of muscle surfaces in late stage 17 embryos of wildtype (wt) or homozygous for *lanA^9.32^* (*lanA*) or for *Df(2L)Exel7022* (*vkg dcg1*). **G,G′**) Neuromuscular bouton profile with synapses in a *Df(2L)Exel7022* mutant embryo showing a reduced adhesion index; see Fig. 1 for explanations of symbols and Fig. 2 for statistical validation of the NMJ phenotypes. Scale bar in A represents 70 µm in A and B (inset 2.5 fold magnified), 45 µm in C, 500 nm in D–G, and 170 nm in G′.

Alternatively, Laminin A could contribute to BM adhesion through its acknowledged roles in BM assembly [60]. Thus, Laminin A might mediate the incorporation of other extracellular matrix proteins into BMs which, in turn, could act as the *bona fide* ligands for cell surface receptors. We therefore tested the molecular composition of BMs in wildtype and *lanA^9.32^* mutant embryos at late stage 17, using specific antibodies against two BM-constituents: the heparan sulfate proteoglycan Perlecan and the small linker molecule Nidogen [51]. In wildtype embryos stained for Laminin, Nidogen or Perlecan, the contours of all tissues (such as muscles, trachea, nerves and CNS) were stained homogeneously, indicating that the label was aligned with surface-attached BMs (Fig. 4D–F). In *lanA^9.32^* mutant embryos, Laminin staining was abolished, whereas Nidogen and Perlecan revealed a sheet-like stain that appeared less homogeneous and no longer displayed clear tissue contours (Fig. 4G–I). These observation were consistent with the ultrastructural finding that BMs are detached from muscle surfaces in Laminin-deficient embryos (Fig. 5E) [28]. In conclusion, our findings strongly suggest that Laminin A-deficient BMs maintain essential other extracellular matrix proteins. Therefore, our data do not support a model in which Nidogen and/or Perlecan serve as the ligands that mediate BM attachment to cell surfaces.

### Partial NMJ detachment is reproduced by loss of type IV collagens

Besides Laminin, type IV collagens are the other class of structural key components of BMs [60]. We hypothesised that removal of these collagens should structurally weaken BMs and provide an alternative strategy to reveal potential mechanical roles of BMs at NMJs, without abolishing possible direct functions of Laminin A in the synaptic cleft.

The *Drosophila* genome harbours three genes for type IV collagen polypeptides, only two of which (Cg25C and Viking/Vkg) are distributed to BMs throughout the body [51]. Of these we assessed the distribution of Vkg by using an isoform genomically tagged with GFP (Vkg::GFP). Vkg::GFP displayed a homogeneous localisation pattern that highlighted prominent tissue contours and was indistinguishable from Laminin, Nidogen and Perlecan stainings (Fig. 5A). Ultrastructural analyses of *vkg^k00236^* loss-of-function mutant embryos did not show any obvious phenotypes (quantified in Fig. 2F). However, BMs of embryos lacking both type IV collagens (*Df(2L)Exel7022* mutant embryos) appeared fragile and discontinuous, but remained closely attached to all cell surfaces (Fig. 5F, G). When staining these embryos with anti-Laminin antibodies, we found a fuzzy localisation that was closely associated with cell surfaces (Fig. 5B, C), consistent with the finding that Laminin A is required for BM anchorage. This observation was in agreement with recent reports [61]. It was clearly supported by our ultrastructural findings that BMs are highly fragmented but maintain surface contact in late stage 17 *Df(2L)Exel7022* mutant embryos (Fig. 5F).

Importantly, NMJ adhesions were reduced to ca. 30% in *Df(2L)Exel7022* mutant embryos, whereas all other structural features of NMJs appeared normal (Figs. 2F and 5G). Therefore, removal of type IV collagens produced a loss of neuromuscular adhesion phenotype strikingly similar to that of Laminin A-deficient embryos, most likely by reducing the mechanical strength of BMs consistent with recent reports [61]. Since collagen IV deficiency did not affect Laminin A localisation, these data strongly suggested that BMs provide mechanical support to neuromuscular adhesion in *Drosophila* embryos by tying boutons onto muscle surfaces, whereas direct roles of Laminin A in the synaptic cleft seem dispensable for NMJ adhesion. Therefore, the BM is a major, previously unappreciated factor that essentially supports NMJ adhesion.

## Discussion

Synaptic CAMs and cleft matrices are crucial players for many aspects of synapse formation, maintenance and plasticity, and they are implicated in numerous neurodevelopmental, psychiatric and neurodegenerative disorders in humans [11], [12]. Redundancy between CAMs is considered a major obstacle to their detailed analysis [62]. Therefore, we used the genetically amenable NMJ of *Drosophila* aiming to crack its adhesion code and establish minimum adhesive conditions in which contributions of single classes of adhesion factors can be investigated in great detail. Unfortunately, even at this relatively simple synaptic contact, NMJ structure and adhesion were astonishingly resistant to genetic manipulation of CAMs. However, two key statements can be deduced. First, our data strongly suggest that the regulatory genetic networks that establish reproducible NMJ adhesions of *Drosophila* embryos are robustly buffered against loss of whole classes of CAMs (in particular classical cadherins, neurexins, 7-transmembrane cadherins, syndecans, integrins and dystroglycan) and the loss of all GluRs. Either these factors are irrelevant for NMJ structure, or the degree of redundancy across different CAM classes is larger than anticipated. Second, Laminin A-dependent BM-to-plasma membrane attachment (rather than Laminin A function within the neuromuscular cleft) contribute to NMJ adhesion in the embryo. In the following, these outcomes are discussed in greater detail.

### Explaining the role of Laminin A and surface attachment of BM

**Figure 6:**
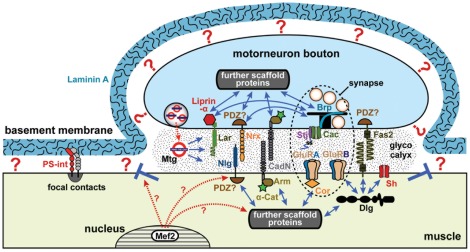
A model view of the embryonic *Drosophila* NMJ. In late *Drosophila* embryos, presynaptic motorneuronal boutons (blue) are attached with half of their surfaces to muscles (beige), and synapses (dashed ellipse) are assembled at these neuromuscular cell-cell contacts. Neuromuscular synapses contain presynaptic active zones with key components such as the scaffolding protein Bruchpilot (Brp) or the Cacophony (Cac) calcium channel including its associated subunit Straightjacket (Stj) [25]. Postsynaptically, neuromuscular synapses contain clusters of GluRs composed of the three obligatory C, D and E subunits and the variable A and B subunits. For most CAMs, such as Leukocyte-antigen-related-like (Lar) [107], Neuroligins (Nlg) [110], [111], Neurexins (Nrx; as mentioned in text), classical cadherins (CadN; as mentioned in text), it remains to be clarified whether they localise within synapses or extra-synaptically; for Fasciclin2 (Fas2) peri-synaptic localisation has already been reported [112]. All these components are interlinked through intracellular scaffolds. Discs large (Dlg) selectively stabilises GluRB receptors at the synapse, but also anchors Shaker potassium channels (Sh) or Fas2 [26], [113]. The band 4.1 superfamily protein Coracle (Cora) interacts with the carboxy-terminus of GluRIIA but not GluRIIB [114], but has likewise been shown to interact with Nrx-IV in other cellular contexts [115]. Links of the Lar-associated scaffold protein Liprin-α to Brp, or of Nrx-IV to Brp have been explained elsewhere [25]. Many more interactions with further scaffold proteins on both sides of the junction are to be expected. The glycocalyx (stippled area) within the synaptic cleft forms a third scaffold established through the linkage of carbohydrate-side chains, often mediated through lectins, such as Mind-the-gap (Mtg). BM links in a Laminin A-dependent manner to cell surfaces through yet unidentified receptors (?), although PS-integrin-mediated Laminin A-independent adhesion at focal contacts has been described [28]. BM is likely to compete with motorneuronal terminals for muscle surface, and BM adhesion needs to be excluded from neuromuscular adhesions (blue T) [116]. Proteins downstream of the Mef2 transcription factor are likely to contribute to this process, as is suggested by complete loss of NMJ adhesion in *mef2* mutant embryos [70].

A distinction between embryonic and larval *Drosophila* NMJs is the complete submersion of larval presynaptic terminals into the muscle, whereas embryonic terminals still sit on the muscle surface and are overlaid by BM (Fig. 6) [23], [24]. Our results strongly suggest that this presence of BMs in the embryo is of high importance, since it lends mechanical support to NMJ adhesion. Like cells in culture (which tend to be rounded if not adherent but flat when adherent), CAM-mediated adhesion is likely to be required to deform the rounded presynaptic boutons and establish extended contacts with the straight muscle membrane (the stiffness of which is likely high in stretched muscles). Our data suggest that the overlaying BMs enhance or facilitate this effect through mechanical support. When BMs loose their physical strength and/or adhesion (upon loss of type IV collagens or Laminin A), NMJ adhesion indices are significantly reduced and likely to be dependent primarily on CAM-dependent forces.

An obvious enigma is the receptor requirement for the Laminin A-dependent cell surface attachment of BMs (red “?” in Fig. 6). Our data show that it can not be explained through any of the major laminin receptor classes alone, nor is it likely to depend on the constituent BM components Nidogen or Perlecan. One possibility is redundancy between the different Laminin receptors or even with the receptors for other BM proteins. Similarly, in vertebrates, “genetic perturbation studies have failed to support a specific receptor as the sole anchor for BM assembly in animal tissues” [60]. For example, in the mammalian epidermis Laminins link to α6ß4 and α3ß1 integrins as well as to transmembrane Collagen XVII [63], [64]. At vertebrate NMJs, Laminins bind to integrins, dystroglycan, the immunoglobulin superfamily CAM Lutheran/Bcam as well as calcium ion channels [19]. Of the unconventional Laminin receptors mentioned here, transmembrane collagens have not been reported in *Drosophila*, transmembrane channels have so far not been implicated in extracellular matrix interactions, and no homologue for the immunoglobulin superfamily CAM Lutheran/Bcam has been reported (although it might be hidden amongst the wide range of immunoglobulin transmembrane receptors encoded by the *Drosophila* genome) [29]. Obviously, the situation is expected to be less complex in *Drosophila* than in vertebrates, and once the key players of BM anchorage are identified this will open up promising opportunities to understand the fundamental principles of BM regulation and its contributions to developmental and disease processes.

Notably, our findings indicate that Laminin A or type IV collagens have CAM-independent functions in embryonic NMJ adhesion. Therefore, future studies on adhesion in this model need to consider including loss of function of these proteins or their receptors. So far, our first attempts combining the *lanA^9.32^* mutant allele with Cadherin- or GluR-deficient conditions or with further single mutations (*klg^EY226^*, *tutl^k14703^, Sdc^23^*), have not yet delivered a precedent demonstrating putative roles of BMs in masking adhesion phenotypes (Figs. 1H and 2C, D; see details in the Fig. 2 legend). Instead, they provide a further argument in support of CAM redundancy, as discussed below.

### Explaining the immunity of neuromuscular adhesions to loss of CAM functions

Even in the absence of structural support through the BM, neuromuscular contacts and synapses are still formed and maintained, though with significantly reduced adhesion indices. The only sensible explanation for this phenomenon is that the various CAMs localising at embryonic NMJs perform roles in adhesion and differentiation. Yet, our extensive studies with loss-of-function of whole classes of CAMs in the presence or absence of BM support failed to reveal obvious phenotypes.

These results could mean that the tested CAMs are irrelevant for the structure of the embryonic *Drosophila* NMJ. However, given the demonstrated presence of all these factors at the neuromuscular contact, we favour the view that CAMs show an enormous degree of functional redundancy. To explain this phenomenon, we propose a multi-adaptor principle based on the rationale that different classes of synaptic CAMs are molecularly and functionally interlinked through three scaffolds: one scaffold on the presynaptic side, one on the postsynaptic side and one in the synaptic cleft (Fig. 6). Thus, as proposed for vertebrate synapses, intracellular linker molecules at pre- and postsynaptic sites form sub-membraneous scaffolds that are associated with the cytoplasmic domains of CAMs and other transmembrane molecules [37], [65]. Also in *Drosophila*, CAMs are linked to sub-membraneous scaffolds, and different CAM classes can even have common binding partners (see examples in Fig. 6) [25], [66]. The pre- and postsynaptic scaffolds are linked across the synaptic cleft through the extracellular domains of CAMs [8]. Extracellular domains of CAMs and other synaptic transmembrane proteins are embedded in a third scaffold within the synaptic cleft, called the glycocalyx [51] (Fig. 6). This glycocalyx consists of sugar side chains of extracellular proteins and protein domains, interlinked essentially through lectins. Its importance is best illustrated by NMJ phenotypes caused by loss-of-function of the lectin Mind-the-gap (Mtg). Loss of Mtg causes severe reduction of electron dense cleft material, of GluRs and of several postsynaptic intracellular scaffold proteins [67].

We propose that these three interlinked scaffolds provide a multi-adaptive and highly robust matrix, the formation of which can be simultaneously triggered by different neuromuscular CAMs, and therefore leads to reliable synaptic maturation even in the absence of whole CAM classes. Such a scenario would be in agreement with observations in mammalian studies. For example, expression of either neuroligin or synCAM in non-neuronal cells is sufficient to attract presynaptic terminals and induce the entire assembly of their presynaptic machineries [8], [9]. *Vice versa*, expression of neurexins in non-neuronal cells is sufficient to attract dendrites and induce postsynaptic receptor fields [68].

In this scenario, the clustering of GluRs into postsynaptic fields is absolutely dependent on contact formation with the presynaptic terminal [69]. This is different for presynaptic active zones. Normally, active zones assemble with high preference at neuromuscular adhesions (most likely through the favoured interaction with presynaptic CAMs or scaffold components), but they still assemble as structurally normal units on neuronal surfaces in the absence of neuromuscular adhesion [70] (Fig. 1). Similarly, orphan presynaptic sites are seen in primary cultures of both *Drosophila* and mouse neurons [71], [72]. This synapse-independent capacity of active zone assembly may be explained through their ability to pre-assemble already within transport vesicles [73].

### Concluding remarks and perspectives

Given the enormous abundance of CAMs at NMJs, their involvement in synaptic adhesion processes *in vivo* is inevitable, but work in vertebrate/mammalian models revealed their enormous resistance to loss-of-function studies likely due to a high degree of redundancy. Our data strongly suggest that such redundancy also exists at the molecularly far simpler invertebrate model synapses, and likely reaches across different CAM classes. However, our data also demonstrate how the availability of a wide range of genetic tools in *Drosophila* and their application through combinatorial fly genetics can be used to obtain an understanding of the mechanisms and components that underpin synaptic adhesion. A good candidate gene to be considered for future studies is the lectin Mtg with its aforementioned roles during the structural organisation of NMJs [51]. Further good candidates are the proteins known to regulate synaptic spacing and bouton formation, such as Straightjacket (the accessory subunit α2δ3 of presynaptic Ca^2+^ channels; Fig. 6) or the cytoskeleton-associated Ankyrins and Spectrins [74]–[77]. Apart from structural analyses of such mutant combinations, future studies should also consider biophysical approaches, such as atomic force analyses, which might provide more direct and sensitive readouts for adhesion strength than the adhesion index used in this study. Notably, any insights gained in the fly are likely to have further reaching implications, based on the experience that principal molecular mechanisms tend to be conserved between *Drosophila* and mammals [78].

## Materials and Methods

### Electron microscopy

Embryos were collected for 1 hr, fixed at late stage 17 (i.e. the time of hatching) [79] as judged by a number of unequivocal stage indicators (filled trachea, dark head skeleton and dense white Malphigian tubules). Mutant embryos were selected using green fluorescent balancers with *Krüppel*- or *twist-Gal4* insertions, as available from the Bloomington stock collections. Glutaraldehyde fixation, staining and embedding protocols are described in detail elsewhere [80]. Imaging was performed on a FEI Tecnai 12 Biotwin transmission electron microscope via either the FEI film camera or a GATAN Orius SC1000 digital camera.

### Immunohistochemistry

Late stage 17 embryos were flat-dissected, fixed and stained following protocols described in detail elsewhere [80]. Antibodies used in this study include: anti-CadN (DN-EX #8, rat, 1∶5, from DSHB) [45], anti-laminin (rabbit, 1∶500, kindly provided by Stefan Baumgartner) [81]; anti-Armadillo/ß-Catenin (N2 7A1, mouse, 1∶5, DHSB) [82]; anti-Perlecan (mouse, 1∶1000, kindly provided by Stefan Baumgartner) [83]; anti-Nidogen (rabbit, 1∶1000, kindly provided by Stefan Baumgartner) [84]; anti-HRP (goat, 1∶50, Jackson Immuno Research). Measurements of minimal, maximal and mean grey values (Fig. 3) were performed using Image J software.

### Fly stocks (all nomenclature according to www.flybase.org)

The following alleles were used: ***CadN^M19^*** (*Cadherin-N*) carries a nonsense mutation at the proximal end of the extracellular domain, leading to complete loss-of-function, a virtually protein-null condition in western blots, embryonic lethality with phenotypes as strong as those of deficiencies uncovering the area [45]; ***CadN-CadN2(Δ14)*** is a small deletion that removes the entire *CadN2* locus (*Cadherin-N2*) and the first half of *CadN* [40] and, accordingly, CadN staining is undetectable in the CNS and at NMJs of *CadN-CadN2(Δ14)* mutant embryos at late stage 17 (Fig. 3); ***stan^192^*** *(starry night, flamingo)* is a genetic null allele [85], [86]; ***Nrx-1^Δ83^*** (*Neurexin-1*) is a small deletion that removes the extracellular domain and transmembrane domain of Neurexin-1, causing complete loss of protein in Western blots [35]; ***Nrx-IV^4304^*** (*Neurexin-IV*) is a protein-negative null mutant [87] generated by EMS-mutagenesis [33] which is commonly used for work on Nrx-IV (www.flybase.org); ***klg^EY226^*** (*klingon*) deletes the entire ORF and no transcripts are detected in embryos [88], [89]; ***tutl^k14703^*** (*turtle*) is a hypmorph allele with demonstrated functions in the CNS and PNS, already in the embryo [90]–[92]; it was the strongest available mutant allele when our experiments were performed; ***GluRIIC^1^*** **( =** ***GluRIII^1^*** **)** (*Glutamate receptor IIC*) is a null allele causing embryonic paralysis [52], [93]; ***Dg^043^*** and ***Dg^086^*** (*Dystroglycan*) carry stop codons in the middle and near the start of the gene, leading to truncations of the protein [94]; ***Sdc^97^*** and ***Sdc^23^*** (*Syndecan*) are small deficiencies removing transcription start, first exon and parts of the first intron of *Sdc*; embryos homozygous for these alleles fail to produce *Sdc* transcripts or protein and display clear mutant phenotypes [95]; all *sdc* and *dg* mutant embryos analysed in our studies were obtained from homozygous mutant mothers to exclude the presence of maternal contribution; ***mys^XG43^*** (*myospheroid*, ß_PS_-integrin) is an EMS-induced null allele removing all protein in embryos; in complementation tests with hypomorphic alleles, *mys^XG43^* behaves like a deficiency [96]; ***ßInt-v^1^*** (*ß^v^* integrin) is a 1431 bp deletion that removes the start of translation and 69 codons of ßInt-v, including the signal peptide; *ßInt-v^1^* mutant flies are viable and fertile, without obvious morphological defects [97]; ***vkg^k00236^*** (*viking*, collagen-IVα2) is a strong hypomorphic or loss-of-function allele that has been demonstrated to produce mutant phenotype in embryos [98], [99]; ***Df(2L)Exel7022*** (Bloomington #7794) is a deletion uncovering the loci of *vkg* and *Cg25C* (collagen-IVα1) [100]; ***Lan^9.32^*** (*Laminin A*; α-laminin) is a null allele caused by deletion removing at least 370 bp of translated sequence at the N terminus of the protein [101]; ***Df(3L)Excel8101*** (Bloomington #7928) is a small deficiency uncovering the lanA locus (http://flybase.org/reports/FBab0038167.html); ***lanB1^DEF^*** *(Laminin B1, ß-laminin)* is a small deficiency uncovering the entire *lanB1* gene and the 5′UTR of the adjacent gene *CG72143* [102]; **vkg::GFP** (*vkg^G454^*) is the FlyTrap line *G00454* (courtesy of E. Martin-Blanco) [103].
